# Perinatal complications associated with preterm deliveries at 24 to 33 weeks and 6 days gestation (2011- 2012): A hospital-based retrospective study

**Published:** 2015-11

**Authors:** Maryam Khoshnood Shariati, Zohreh Karimi, Mahroo Rezaienejad, Azita Basiri, Farahnaze Torkestani, Soraya Saleh Gargari

**Affiliations:** 1 *Neonatal Unit, Mahdiyeh Hospital, Shahid Beheshti University of Medical Sciences, Tehran, Iran.*; 2 *Feto-Maternal Unit, Mahdiyeh Hospital, Tehran, Iran.*; 3 *Department of Obstetrics and Gynecology, Mahdiyeh Hospital, Shahid Beheshti University of Medical Sciences, Tehran, Iran.*; 4 *Infertility and Reproductive Health Research Center, Shahid Beheshti University of Medical Sciences, Tehran, Iran.*

**Keywords:** *Premature rupture of fetal membranes*, *Pre-eclampsia*, *Infant death*, *Respiratory distress syndrome*, *Necrotizing entrocolitis*

## Abstract

**Background:**

Morbidity and mortality of preterm babies are important issues in perinatal medicine. In developed countries, preterm delivery is the cause of about 70% of mortality and 75% of morbidity in the neonatal period, respectively.

**Objective:**

The aim of this study was to determine the risk factors for preterm labor and the outcomes, in terms of perinatal mortality and morbidity at the time of discharge home, among preterm infants at less than 34 weeks gestation.

**Materials and Methods:**

A retrospective study was conducted and all infants with a gestational age of 24 to 33 weeks and 6 days who were born from November 1^st^, 2011 to March 31, 2012 were enrolled in this study.

**Results:**

From 1185 preterm infants were born during this period, 475 (40.08%) infants with less than 34 weeks gestational age were included in the study. Our study showed the major obstetrical risk factors for preterm labor were as follows: preeclampsia (21%), premature rupture of membranes (20.3%), abruption of placenta (10%), and idiopathic cases (48.7%). The neonatal mortality rate in less than 34 weeks was 9.05%. Significant perinatal morbidity causesd in less than 34 weeks were as follows: sepsis (46.94%), respiratory distress syndrome (41.47%), patent ductus arteriosus (21.47%), retinopathy of prematurity (3.57%), necrotizing entrocolitis (1.68%), intra-ventricular hemorrhage (9%), and broncho-pulmonary dysplasia (0.84%).

**Conclusion:**

Preterm birth is associated with adverse perinatal outcome. This situation needs to be improved by directing appropriately increased resources for improving prenatal health services and providing advanced neonatal care.

## Introduction

Prematurity is defined as a birth that occurs before 37 completed weeks (fewer than 259 days) of gestation. Worldwide, the estimated rates of the preterm birth is about 11 percent ranging from 5 percent in parts of Europe to 18 percent in parts of Africa), and about 15 million children are born preterm each year (ranging from 12 to 18 million) (1, 2). 

Nearly 70 to 80 percent of preterm births happen without apparent external influence and are related to preterm labor (40- 50%) or preterm rupture of membranes (20- 30%). The rest of preterm births (i.e. 20-- 30%) are due to medical reasons related to maternal or fetal problems (3). The mortality risk from prematurity is greater in low- and moderate-income countries. In a systematic review, pooled data from studies conducted in Latin America, Africa, and Asia reported that preterm infants (gestational age less than 37 weeks) had a 6.8-fold increase in neonatal death compared with term infants Relative Risk (RR) 6.82, 95% CI 3.56-13.07) (4). Preterm delivery can subject children with serious health problems. 

The victims include nearly 45% of children with cerebral palsy, 35% of those with visual impairment and 25% of cognitively and aurally impaired children. Complications due to preterm delivery can be transient or lasting. The transient complications, such as cardiovascular complications, are limited to neonatal period while the long-term complications, such as cerebral palsy, will be with patients even after they leave the intensive care unit (5). 

The growing number of preterm neonates in tertiary centers substantiates the importance to better understand and medically approach preterm infants. Short-term and long-term evaluation, monitoring and follow up of preterm infants are needed to optimize neonatal care and improve human health status. The aims of this investigation were to evaluate the rate of mortality and discharge home morbidity of preterm infants with gestational age at less than 34 weeks and to analyze risk factors associated with preterm birth.

## Materials and methods

In this cross sectional study, medical records of women who had attended the obstetric ward of Mahdieh Hospital, Shahid Beheshti University of Medical Sciences from November 1st, 2011 to March 31, 2012 were studied. 

Our inclusion criteria were: having a singleton pregnancy and having delivered a live infant in a time span from 24 to 33 weeks and 6 days gestational age. Exclusion criteria were: having incomplete data in their medical files. 

Demographic characteristics including age, gravidity, medical history, and clinical information regarding antenatal care, gestational age at delivery, obstetric and postpartum complications, mode of delivery, immediate neonatal outcomes, 5-minute Apgar score, steroid consumption before delivery, surfactant therapy, and mechanical ventilation after birth and early or late neonatal death were retrieved from the medical records. Gestational age was based on the last menstrual period, ultrasound or both. 

Preterm delivery was defined as delivery before complete 37 weeks gestation. Premature rupture of membranes was defined as rupture of the amniotic membranes before the onset of labor. Fetal growth restriction or small for gestational age was diagnosed if the actual birth weight was below the 10^th^ percentile for gestational age. Prenatal data including neonatal birth weight, intrauterine fetal death, still birth, neonatal intensive care unit admission and information about the demographics and the outcome of all live born infants from 24 to 33 weeks and 6 days gestational age, born at Mahdieh Hospital were collected. Data were collected on all infants until death or discharge home. Mortality rates were calculated for all infants born alive. Morbidity rates and treatments of those infants admitted to a neonatal intensive care unit, and were shown to have the following diagnoses were collected: intraventricular hemorrhage (IVH), based on the most severe ultrasound result during the hospital stay using the classifications defined by Papile *et al* (6); retinopathy of prematurity using the international classification published by the committee for the classification of retinopathy of prematurity (7); bronchopulmonary dysplasia (BPD) defined as an oxygen requirement at 36 weeks gestational age according to the Eunice Kennedy Shriver National Institute of Child Health and Human Development (NICHD) consensus conference paper (8); necrotizing enterocolitis defined as clinical signs (abdominal distension, bilious aspirates and/or bloody stools) confirmed by radiographically visible intramural gas or at laparotomy (Bell stage 2 and 3) (9); patent ductus arteriosus which was symptomatic and required indomethacin or surgery; sepsis is defined both clinically and/or microbiologically, by positive blood and/or cerebrospinal fluid cultures (10, 11); respiratory distress syndrome (RDS) was diagnosed in babies with respiratory distress (requirement for oxygen supplementation due to tachypnea, grunting, nasal flaring, retractions and/or cyanosis) together with chest X-ray and blood gas analysis (12). 

The study was approved by the Ethics Committee of Shahid Beheshti University Of Medical Sciences, Tehran, Iran.

**Statistical analysis**

It is just a descriptive study with no comparison.

## Results

Totaly, 475 preterm infants (gestational age < 34 weeks) (15.4% of all newborn infants) were delivered to 475 mothers from November 1^st^, 2011 to March 31, 2012. The mean age of mothers were 28.9±15 years (range: 15-46 years). The occurrence of pregnancy complications diagnosed before delivery was: preeclampsia (19.7%), premature rupture of membranes (22%), abruption of placenta (8.8% and 49.5% idiopathic. Infant morbidity and mortality rates by gestational age at birth are given in table I.

Antenatal steroid treatment was administered to 43.6% of the mothers of live born infants with gestational age below 34 weeks.

Table II indicates the rate of administration of antenatal steroids, surfactant treatment, nasal continuous positive airway pressure (CPAP) and advanced resuscitation infants according to gestational age. When birth characteristics of newborns were checked it showed for very preterm and moderate births mean gestational age were 29 w 6 days and 33 w 1day respectively. Also it was found that Mean weight for very pretem infants was 1235 gr and 2105 gr for moderate pretem births respectively.

Neonatal mortality rate in infants between 32-33 weeks and 6 days was 2.16% and in babies at less than 32 weeks was 12.45% during the study period (Table III).

**Table I:** Neonatal complications of very preterm (n=247) and moderate preterm infants (n=228)

**complications**	**Very preterm n (%)**	**Moderate preterm n (%)**
Respiratory distress syndrome	87 (35.4%)	110 (48.24%)
Retinopathy of prematurity	15 (6.1%)	2 (0.87%)
Patent ductusarteriosus	60 (24.3%)	42 (18.4%)
Bronchopulmonary dysplasia	4 (1.6%)	0 (0%)
Necrotizing entrocolitis	5 (2.02%)	3 (1.3%)
Sepsis	98 (39.7%)	125 (54.8)
Intraventricular hemorrhage	3 (9%)	2 (0.87)
Death	37 (15%)	6 (2.6%)

Very preterm include infants who are delivered less than 32 weeks gestational age and moderate preterm encompasse babies born from 32 to 33 weeks and 6 days gestational age

**Table II:** The rate of administration of antenatal steroids and infant surfactant treatment, nasal CPAP, and advanced resuscitation were used according to gestational age

**Treatment**	**Very preterm (N=247)** **n (%)**	**Moderate preterm (N= 228)** **n (%)**
Antenatal Steroid	90 (36.4%)	119 (52.2%)
Surfactant therapy	74 (30%)	40 (17.5%)
Nasal CPAP	113 (37.7%)	129 (56.5%)
Advanced resuscitation	63 (25.5%)	26 (11.4%)

CPAP: continuous positive airway pressure

**Table III:** The ferequncy of death according to infant age

**Death**	**Very preterm (N=247)** **n (%)**	**Moderate preterm (N= 228)** **n (%)**
Death in delivery room	7(2.8%)	1(0.43%)
Death in first 24 hours of life	12(4.85%)	1(0.43%)
Death in first week	5(2%)	2(0.87%)
Death from the 1^st^ week until 28 days of life	5(2.8%)	1(0.43%)

## Discussion

This study was conducted to assess risk factors for preterm birth and perinatal outcome in terms of perinatal mortality and morbidity for infants born at less than 34 weeks gestation. As known premature births are increasing in recent years and the highest rate of mortality and morbidity are seen in this age. Our results showed that preterm babies represented about one-fifth (23%) of all newborn births in our hospital; among these 40.08% were less than 34 weeks gestation. Significant obstetrical risk factors for preterm labor were preeclampsia (21%), premature rupture of membranes (20.3%), abruption of placenta (10%) and idiopathic cases (48.7%). These results were consistent with other studies' findings (3).

In another report conducted in Shiraz, the rate of preterm delivery was significantly higher as compared to the results obtained in this study. This may be due to the improvement of prenatal care services during the last decade in Iran (13).

The neonatal mortality rate in infants at less than 34 weeks was 9.05%. In infants with gestational age of 32-33 weeks and 6 days, the mortality rate was 2.6%. In our study, while no mortality was reported in infants with the same age by Bastek et al study (14). In our study, for infants with gestational age less than 32 weeks, the mortality rate was 15%, that is similar to Larroque *et al* (14%) and Torres *et al* report (16%) (15, 16). While in Stoelhorst *et al* study this rate was shown to be 11% (17). Higher infant mortality in the present study could have been arisen from a low percentage of pregnant mothers who received antenatal treatment with corticosteroid (36% versus 73%) (17).

Of course, the lower level of care in neonatal intensive care unit may also contribute to the higher incidence of our mortality. In the present study, the most common cause of death in infants less than 28 weeks is was sepsis (27.9%), RDS (23.3%) and pneumothorax (19.2%). In Stoelhorst *et al* study, RDS and cerebral complications have been the causes of 45% and 24% of deaths respectively (17). Our results demonstrated advanced resuscitation rates in the delivery room in infants with gestational age less than 28 weeks, 28-29 weeks and 6 days, 30-31 weeks and 6 days were 42.2%, and 18.5%, 18.9%, respectively. It can be a major contributing factor in increasing mortality.

In infants with gestational age less than 32 weeks, IVH rate was 9 % (7.5% grade I, II and 1.5% grade III, IV). Compared to Stoelhorst *et al* and Bastek *et al* findings, these differences may be related to high frequency of RDS in Stoelhorst *et al* study and high prevalence of chorioamnionitis in Bastek *et al* study (14, 17). Our results showed in babies with gestational age of 32-33 weeks and 6 days gestation IVH rate was 0.87%, while Escober and coworkers reported lower rate of IVH (0.6% in babies with 30-31.6 weeks and 0% in infants with 32-33.6 weeks gestation). This was perhaps due to higher steroid treatment of infants in Escober *et al* study (18).

In the current study, nearly 55% of infants with gestational age ranged 32-33 weeks and 6 days and 40% of babies with gestational age less than 32 weeks had a clinical or positive culture of sepsis. This is higher than incidence of sepsis in the Stoelhorst *et al*'s study (28%). This could have resulted from different criteria that we used for the diagnosis of sepsis. In Stoelhorst *et al.* study the diagnosis of sepsis was based on positive blood culture, whereas in our study, the diagnostic criteria were clinical signs and (or) positive blood culture (17). The incidence of sepsis was 43.4% in infants with gestational age 32-33 weeks and 6 days in Bastek *et al*'s report which demonstrates the need for extra care in our hospitals (14).

The occurrence of RDS was 35.4 % in infants with gestational age at less than 32 weeks in our study which was lower, compared to Stoelhorst *et al.* report (60%) and Torres *et al.* study (64%) (16,17). This is justified by a higher incidence of our mortality in the delivery room or in the first few hours of birth before developing symptoms of respiratory distress syndrome or more infants with small for gestational age or ethnicity differences. In our study, 30% of infants less than 32 weeks received surfactant treatment, which was lower than the Stoelhorst and coworkers study (42% of cases received surfactant) (17). Basteke *et al.* reported a 41% rate of RDS in infants with gestational age between 32 to 33 weeks of gestational age that was lower than our finding (48.24). This may be related to the high prevalence of chorioamnionitis in Bastek *et al.* (i.e. 37.7% versus 0% in our study) that accelerated maturation of the lungs. 17.5% of our infants between 32 and 33 weeks and 6 days have received surfactant, which was not much different compared to Basket *et al*. study (18%). 17.5% of our babies through 30 and 33 weeks and 6 days received surfactant treatment that was similar to Bastek *et al* report (18%) (14). But more than 24.6% of babies with the same gestational age received treatment in Scober *et al.* study (18).

In the present study, the incidence of patent ductus arteriosus in infants with less than 32 weeks was 24.3% that was slightly higher compared with other study (26%) (17). It may be due to ethnicity differences or prophylactic use of indomethacine in our center.

In addition, in this study the retinopathy of prematurity rate was 6.1%, 0.87%, and 2.1% among infants with less than 32, 32-33 and 6 days and 30-33 weeks and 6 days of gestation, respectively, that was greater than that of Escober and coworkers' study in age groups of 30-33 weeks and 6 days (0%) (18). A systematic approach should start from the delivery room in order to reduce the rate of this morbidity. In our study, the frequency of broncho pulmonary dysplasia was 1.6% among infants at less than 32 weeks of gestation. In the Stoelhorst *et al.* report the prevalence of this diagnosis was 19% and the frequency of this morbidity was reported 6.7%. Escober and coworkers reported this finding with a frequency of 1% among infants 30-31 weeks and 6 days and 32-33 weeks and 6 days of gestation (17, 18). This discrepancy may be due to greater use of nasal CPAP in our center. In our study, nasal CPAP was used in 43.7% and 56.5% of infants through 30-31weeks and 6 days and 32-33 weeks and 6 days, respectively, while in Escober *et al*s study this rate was 19% among infants. Only 18.9% of our infants received mechanical ventilation versus 45.7% in Escober *et al* study. This discrepancy is acceptable due to the high rate use of nasal CPAP (18).

The prevalence of necrotizing enterocolitis was 2.02% among infants born with gestational age less than 32 weeks and 1.3% among babies born at 32 to 33 weeks and 6 days respectively. Stoelhorst and coworkers reported the necrotizing entrocolitis rates 9% among infants born with gestational age less than 32 weeks. Whereas these frequencies were published 1.2% and 0.7% for infants born through 30-31 weeks and 6 days and 32-33 weeks and 6 days, respectively in Escober *et al.* study and the prevalence of this morbidity was reported 8.2% for infants born through 32 -33 weeks gestation in Basket *et al.* study. This significant difference is acceptable due to better care of infants in Escober *et al.* study and a high percentage of their mother who received treatment with betamethasone. While in comparison with Bastek *et al.* report, it may be justified by high prevalence of chorioamnionitis (37.7%) in Bastek *et al.* study. The high rate of infants mortality before admission in the hospital may be a possible explanation for the difference seen with Stoelhorst *et al* study (14, 17, 18).

regarding the result of this study mortality and sepsis rates of infants at less than 32 weeks is greater than similar studies and rate of RDS, IVH, BPD and necrotizing enterocolitis are less than the other studies. In our study, with increasing gestational age from 28 to 34 weeks, the mortality rate is clearly reduced, but it is not true about incidence of sepsis and respiratory distress. This may be due to pregnancy with premature rupture of membrane expectantly managed until 34 weeks gestational age. This reason is required to be examined in future studies.

This study found that in order to achieve significant reductions in adverse outcomes of preterm infants the following step should be taken. First, the clinicians must focus their efforts on prevention of preterm birth by implementation of effective strategies for identifying the high risk group of preterm delivery in asymptomatic women by sonographic measurement of cervical length at 18 weeks gestational age during anomaly scan and special attention should be placed in the uterine artery PI for prediction of preeclampsia at 12 weeks gestational age during first trimester screening. Second, it is necessary to pay more attention to improve our mothers’ knowledge about the warning signs of pregnancy since a high proportion of high risk mothers, admitted just at the time of delivery, make management of these groups difficult. Third, appropriately increased resources should be considered to meet the needs for care of this high risk babies and greater attention needs to be paid for conducting formal evaluations of the therapies and to follow-up strategies employed in caring of preterm infants. This study has several limitations. First, this is a retrospective study, which may encompass some missing data. Second, the data were collected just from one hospital files which may not be too representative of all maternity hospitals in Iran. Also, the sample size should be increased in further studies.
